# Cardiac Autonomic Nervous System Activation and Metabolic Profile in Young Children: The ABCD Study

**DOI:** 10.1371/journal.pone.0138302

**Published:** 2015-09-22

**Authors:** Tanja G. M. Vrijkotte, Bert-Jan H. van den Born, Christine M. C. A. Hoekstra, Maaike G. J. Gademan, Manon van Eijsden, Susanne R. de Rooij, Marcel T. B. Twickler

**Affiliations:** 1 Department of Public Health, Academic Medical Center—University of Amsterdam, Amsterdam, the Netherlands; 2 Department of Vascular Medicine, Academic Medical Center—University of Amsterdam, Amsterdam, the Netherlands; 3 Department of Epidemiology and Health Promotion, Public Health Service of Amsterdam (GGD), Amsterdam, the Netherlands; 4 Department of Clinical Epidemiology, Biostatistics and Bioinformatics, Academic Medical Center, Amsterdam, the Netherlands; 5 Department of Endocrinology, Diabetology and Metabolic Diseases, Antwerp University Hospital, Antwerp, Belgium; Shanghai Institute of Hypertension, CHINA

## Abstract

**Background:**

In adults, increased sympathetic and decreased parasympathetic nervous system activity are associated with a less favorable metabolic profile. Whether this is already determined at early age is unknown. Therefore, we aimed to assess the association between autonomic nervous system activation and metabolic profile and its components in children at age of 5–6 years.

**Methods:**

Cross-sectional data from an apparently healthy population (within the ABCD study) were collected at age 5–6 years in 1540 children. Heart rate (HR), respiratory sinus arrhythmia (RSA; parasympathetic activity) and pre-ejection period (PEP; sympathetic activity) were assessed during rest. Metabolic components were waist-height ratio (WHtR), systolic blood pressure (SBP), fasting triglycerides, glucose and HDL-cholesterol. Individual components, as well as a cumulative metabolic score, were analyzed.

**Results:**

In analysis adjusted for child’s physical activity, sleep, anxiety score and other potential confounders, increased HR and decreased RSA were associated with higher WHtR (P< 0.01), higher SBP (p<0.001) and a higher cumulative metabolic score (HR: p < 0.001; RSA: p < 0.01). Lower PEP was only associated with higher SBP (p <0.05). Of all children, 5.6% had 3 or more (out of 5) adverse metabolic components; only higher HR was associated with this risk (per 10 bpm increase: OR = 1.56; p < 0.001).

**Conclusions:**

This study shows that decreased parasympathetic activity is associated with central adiposity and higher SBP, indicative of increased metabolic risk, already at age 5–6 years.

## Background

It is currently assumed that the origin of atherosclerosis and diabetes mellitus type II evolve at young age with subtle metabolic and hemodynamic derangements already present at young age[1–3]. In adults, clustering of these metabolic and hemodynamic derangements is known as the metabolic syndrome and is associated with an increased risk of cardiovascular disease and diabetes mellitus type II[4,5]. As there is no current consensus about the delineation of the metabolic syndrome in young children, such cluster of cardiovascular risk factors is actually often referred to as an adverse metabolic profile[6]. Despite this omission in childhood definition, more research in this field is worthwhile as (subtle) metabolic derangements in children may originate health and well-being during later life. Another factor is the coexistent growing epidemic of childhood obesity with concomitant cardiovascular risk factors leading to a subsequent burden for health care systems worldwide.

One of the causes of an adverse metabolic profile could be disbalance in the autonomic nervous systems (ANS) activation. ANS is one of the major adaptors of the stress response (besides the hormonal adaptation mediated by the adrenal gland) and its origin is located in the central nervous system with a peripheral network distributed in a parasympathetic and a sympathetic system. The extent of activity of either the sympathetic or parasympathetic system is associated with the actual stress level, and its net balance influences various metabolic systems[1,7]. It is currently assumed that a long-term increase in stress response, resulting in an unbalanced ANS towards increased sympathetic activity and decreased parasympathetic activity, contributes to obesity, insulin resistance, dyslipidemia and hypertension[8–14]. For example, in a large adult cohort Licht et al. found that increased sympathetic activity and decreased parasympathetic activity were strongly associated with a higher prevalence of the metabolic syndrome and also with its individual components[9]. Both Licht et al.[15] and others[16] showed that this dysregulation of the ANS predicted metabolic abnormalities.

This association has hardly been studied in children, although it is known that chronic high stress levels early in childhood can have permanent negative effects on the development of the brain, and the metabolic and endocrine systems[1]. Recently, Zhou et al. studied 180 non-obese Chinese children at age 9–11 years who received nutrition counselling and found decreased parasympathetic activity, measured by heart rate variability (HRV) indices, with an increasing number of adverse metabolic components[17]. However, their results applied to a selected pediatric population and possible confounding factors were not taken into account. Several small case-control studies examining the association between obesity and ANS activity in childhood have shown generally consistent results regarding lower parasympathetic activity in obese children[18–23], but more conflicting results regarding sympathicovagal balance or sympathetic activity[18–25]. Differences in the methodology, e.g. time vs. frequency domain ANS measures, clinical vs. non-clinical setting and mainly small sample sizes, could explain these differences.

The ABCD cohort is one of the largest community-based birth cohorts in Europe with the inclusion of apparently healthy children aged 5–6 years; its design enables to evaluate whether an altered ANS activity is associated with an adverse metabolic profile already in childhood, taking into account important covariates (such as physical activity, sleep duration and anxiety) known to influence ANS activity[2,18,26]. Therefore, in this cross-sectional analysis, we assessed whether an association exists between changes in sympathetic/parasympathetic activity and their balance with regard to the metabolic profile in children aged 5–6 years.

## Materials/Subjects and Methods

This study is part of the Amsterdam Born Children and their Development (ABCD) cohort study (www.abcd-study.nl). The ABCD study is a prospective community-based cohort study that examines the association between maternal, pregnancy and early life factors and later health and health differences in the offspring. Details of the ABCD study design have been described earlier[27,28]. Approval of the study was obtained from the Central Committee on Research Involving Human Subjects in the Netherlands, the medical ethics review committees of the Academic Medical Center, Amsterdam, the VU University Medical Center Amsterdam and the Registration Committee of the Municipality of Amsterdam. All women provided written informed consent. Parents or caretakers provided written informed consent for the health check of the 5–6 year old children in the questionnaire that was send around two weeks after the child’s fifth birthday. This questionnaire was only send to mothers who initially gave permission for follow-up. This informed consent procedure was approved by the committees of the Academic Medical Center, Amsterdam, the VU University Medical Center Amsterdam and the Registration Committee of the Municipality of Amsterdam.

### Subjects

**Fig 1:**
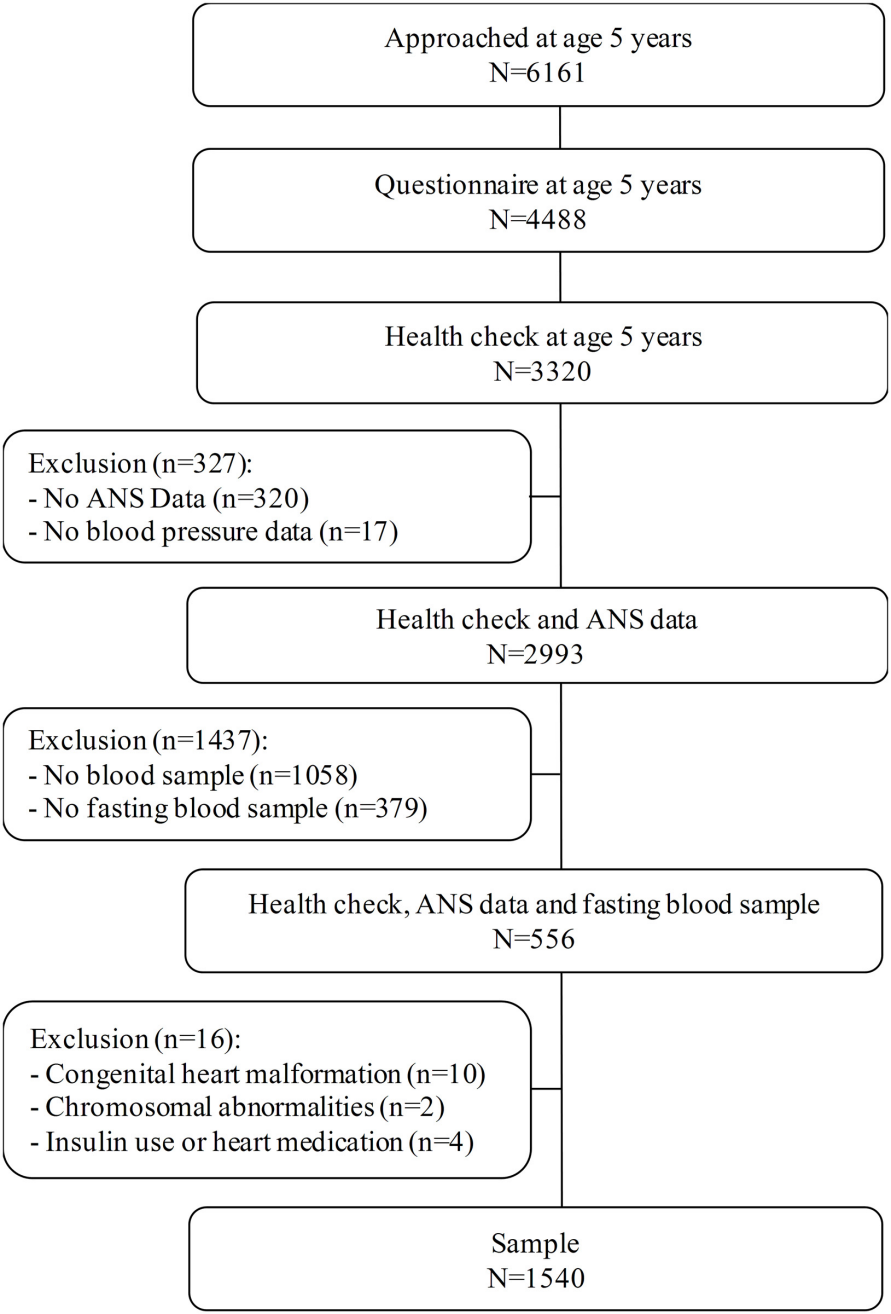
Schematic presentation of the study with all exclusion criteria.

During 2003–2004, 12,373 pregnant women from Amsterdam were invited to participate in the ABCD study. Of these women, 8266 returned the pregnancy questionnaire, which covered socio-demographic characteristics, obstetric history, lifestyle and psychological factors[27]. Of the mothers with a singleton live birth (n = 7863), 6735 gave permission for follow-up (86%). When the children turned 5 years, 6161 mothers received a questionnaire. Attrition was largely attributable to untraceable changes in address, or to migration. The mothers were asked to fill in a questionnaire about their offspring to which 4488 responded (73%). The age 5 health check was performed in 3321 (54%) children of which a subgroup (1935) also agreed to finger stick blood collection. Children who attended the health check were included if a fasting blood sample (n = 1556) was taken. Excluded were children with congenital conditions affecting the cardiovascular system or the autonomic nervous system (n = 10), chromosomal abnormalities (n = 2) and children using medication influencing the autonomic nervous system (n = 4). The final sample consisted of 1540 children. The progression of the study cohort is presented in Fig 1.

### Cardiac autonomic nervous system activity

The children were measured noninvasively by the VU University Ambulatory Monitoring System (VU-AMS version 5fs) during the day between 8:30 am and 16:30 pm. The use, validity and reliability of the VU-AMS have been described previously[8,15,28]. During a standard protocol, three-lead electrocardiograms (ECG) and four-lead impedance cardiograms (ICG) were recorded; the procedure has been described elsewhere[28]. In short, the child was lying down in a supine position for 1 min of stabilization, followed by about 6 min of registration. Next, the child was seated at a table for 1 min of stabilization followed by another 6 min of registration.

The interbeat interval time series was extracted from the ECG signal to obtain heart rate (HR). The software automatically marked inspirations and expirations in the respiratory signal. Respiratory sinus arrhythmia (RSA) was automatically obtained by peak valley estimation as a derivate of parasympathetic nervous system activity[29,30]. As a derivate of sympathetic activity, pre-ejection period (PEP) was used[31]. PEP is the time interval between the onset of the ventricular depolarization (Q wave onset in the ECG) and the time of opening of the aortic valves (B point in the ICG) and was scored manually in large-scale ensemble averages of the impedance cardiograms. To investigate whether the sympathetic and parasympathetic (co-) activity or inhibition is related to an adverse metabolic profile, the cardiac autonomic balance (CAB) and cardiac autonomic regulation (CoAR) were calculated[16]. The CAB was calculated as the difference between normalized values of RSA and PEP[CAB = zRSA-(-zPEP)]. Low values reflect parallel high sympathetic and low parasympathetic control. CoAR was calculated as the sum of the normalized values and low values represent coinhibition (low SNS and low PNS activity) of the two cardiac branches. Summarizing, the outcome measures were HR, RSA, PEP and CAB, CoAR, obtained in both supine and sitting position.

### Metabolic profile

Blood samples were collected by finger stick after an overnight fast in the morning by a validated ambulatory collection kit (Demecal kit: LabAnywhere, Haarlem, the Netherlands) before ANS measurements[32]. Triglyceride (TG), glucose and high-density lipoprotein (HDL) cholesterol were determined [32,. Waist circumference was measured to the nearest mm using a Seca measuring tape, height was measured to the nearest mm using a Leicester portable height measure (Seca, Hamburg, Germany). Waist-to-height ratio (WHtR) was calculated as the ratio of the waist circumference (cm) and the height (cm) which gives a representation of the child’s body size[33]. Systolic and diastolic blood pressure (SBP/DBP) were measured twice (after one test measurement which was discarded) in supine position after 5 min of rest, with the Omron 705 IT (Omron Healthcare Inc, Bannockburn, IL, USA) with a small cuff (arm circumference 17–22 cm) on the non-dominant arm, that is validated in children[34]. If these measurements differed by > 10 mm Hg (SBP or DBP), a third measurement was taken. The SBP and DBP were determined by averaging the two closest measurements per child.

We used WHtR, SBP, triglyceride, HDL and glucose levels as individual components of the metabolic profile. Also, the sum of the metabolic components (first normalized by computing z-scores) was calculated as a clustered metabolic score. HDL z-score was multiplied by -1 to indicate a less favorable profile with increasing values[35]. DBP was not included because this would result in a metabolic score unevenly influenced by childhood blood pressure. More important, SBP is known to be a better predictor of hypertension and an adverse metabolic profile in later life [36]. In addition to the continuous scores, we also defined cut-off values for an adverse metabolic profile according to previous definitions used among pediatric populations[6]: ≤25^th^ percentile for HDL-cholesterol (<1.00 mmol/L), ≥75^th^ percentile for TG (≥ 1.00 mmol/L) and glucose (≥ 4.9 mmol/L). For pre-hypertension in children, cut-offs for SBP and DBP were used according to international classifications, gender and age specific [37]; WHtR is suggested to be unfavorable if ≥ 0.5[33]. Three or more adverse metabolic components were considered to be an adverse metabolic profile.

### Covariates

Covariates included in the analysis were child age (years, continuous), sex, education level of the mother as an index of socioeconomic status (SES; years of education after primary school, continuous), ethnicity, based on the country of birth of the mother (categorized into Dutch, Turkish, Moroccan, Surinamese, other non-Western, other Western), child’s sports participation at a club (categorized into <1 h, 1 h and >1 h per week), child’s screen time (TV and gaming time, hours per day, continuous), hours of sleep per night during school days (categorized into <8, 8–10, 10–11 and >11) and general anxiety (sum score of generalised anxiety subscale from the Preschool Anxiety Scale, continuous[38]). Gestational age (weeks), birth weight (grams), body mass index (BMI in kg/m^2^), waist circumference (WC in cm) and total fat%, obtained by bioelectrical impedance[39], were used to profile the children. Overweight status was based on international cut-offs[40]. Birth weight and gestational age were obtained by the Youth Health Care Registration. Country of birth and education level of the mother from the pregnancy questionnaire. Child’s sport participation, screen time, sleep duration and general anxiety were reported by the mother in the childhood questionnaire.

### Statistical analysis

Baseline characteristics were compared across groups with a different number of metabolic components using ANOVA (continuous) and Chi-square test (categorical). Multivariable linear regression analyses were performed to determine associations between HR, RSA, PEP, CAB, CoAR and the metabolic components. Multivariate model 1 controlled for child’s sex, age, time of the day of the ANS measurement and, in the case of SBP and total adverse metabolic profile, also for the child’s height. In model 2 additional adjustments were made for SES, ethnicity, sports participation, screen time, sleep duration and general anxiety. Subsequently, we conducted logistic regression analysis to explore the association between ANS and increased risk of the individual metabolic components, as well as a clustered profile (≥ 3 out of 5 adverse components).

Analyses were carried out for all ANS measures in both supine and sitting position, independently as well as combined (in one model). However, correlations between supine and sitting values were high (HR:0.89, RSA:0.78, PEP:0.70, CAB:0.78, CoAR:0.77) and both positions provided the same results. Both positions entered in one model revealed that lying down explained the largest part of the variation in the outcome measures and sitting made no significant contribution; therefore only the results in supine position are reported.

The residuals of the linear regression models were visually checked to assess their distribution and homogeneity of variance was assessed with the Levene test. The Hosmer-Lemeshow test was used to assess the goodness of fit of the logistic regression models. TG levels were log transformed to improve the normality of the residuals in the various models. The SPSS version 19.0 (SPSS Inc., Chicago IL, USA) was used to analyze the data.

## Results

### Study population

The children were evaluated at an average age of 5.6 (SD 0.4) years, 49.4% were girls and the ethnicity was predominantly Dutch (75.8%). Compared with children who were approached for the 5-year measurements but were not included (non-response, no permission for a blood sample or did not provide a fasting blood sample; Fig 1; n = 4579), the current subgroup of children were more frequently of Dutch origin, of families with higher SES, had higher birth weight and had mothers who were slightly older (S1 Table). Table 1 presents the children’s characteristics according to the presence of adverse metabolic components. Of all children, 86 (5.6%) children had 3 or more adverse metabolic components; on average, these children were of families with lower SES, spent more time behind a television/computer screen, scored higher on general anxiety, and were slightly older (all p<0.05).

**Table 1:** Sample characteristics.

	Number of metabolic components				
Characteristics	0	1	2	3 or more	p-value
	n = 576	n = 573	n = 305	n = 86	
	37.4%	37.2%	19.8%	5.6%	
	Mean/% (SD)	Mean/% (SD)	Mean/% (SD)	Mean/% (SD)	
**Girls (%)**	49.7	49.2	47.5	55.8	0.600
**Gestational age (weeks)**	39.9 (1.6)	39.9 (1.5)	39.8 (1.9)	39.8 (1.8)	0.501
**Birth weight (grams)**	3492 (523)	3513 (517)	3479 (573)	3487 (552)	0.593
**Education level of mother (Years after primary school)**	10.1 (3.6)	9.9 (3.5)	9.3 (4.2	8.9 (3.6)	0.002
**Ethnicity (Mother) (%)**					0.233
Dutch	75.1	78.4	73.1	68.6	
Turkish	2.1	1.9	3.3	3.5	
Moroccan	3.5	4.0	6.2	7.0	
Surinamese	3.7	2.8	3.3	3.5	
Other non-Western	7.5	8.4	7.9	11.6	
Other Western	8.1	4.5	6.2	5.8	
**Characteristics of child at age 5 years**					
**Age (years)**	5.5 (0.4)	5.5 (0.4)	5.6 (0.4)	5.7 (0.5)	0.015
**Sports participation at a club (%)**					
<1 hour per week	54.7	61.2	65.6	54.3	0.012
1 hour per week	25.3	24.5	18.4	22.2	
>1 hour per week	20.0	14.3	16.0	23.5	
**Screen time (hours/day)**	1.4 (0.9)	1.5 (1.1)	1.5 (1.0)	1.7 (1.1)	0.021
**Hours of sleep week (%)**					0.069
<8 hours per day	1.3	3.1	4.1	0.0	
8–10 hours per day	13.0	14.7	15.8	22.2	
10–11 hours per day	52.4	48.8	48.1	51.9	
> 11 hours per day	33.4	32.4	32.0	25.9	
**Mental health**					
Total anxiety score	6.0 (1.2)	6.0 (1.4)	6.2 (1.5)	6.5 (1.7)	0.003
**Autonomic measures**					
HR (beats/min)	85.9±8.9	86.6±9.5	86.9±10.2	89.5±10.9	0.011
RSA (ms)	125.4±63.8	121.3±58.1	116.1±54.6	111.5± 56.5	0.065
PEP (ms)	70.9±8.8	70.9±8.9	71.0±9.0	71.3±8.6	0.986
CAB	-0.0004±1.5	-0.07±1.4	-0.14±1.4	-0.19±1.4	0.465
CoAR	0.07±1.2	0.01±1.3	-0.09±1.3	-0.19±1.2	0.184
**Metabolic profile measures**					
WHtR	0.45 (0.02)	0.45 (0.03)	0.46 (0.03	0.48 (0.04)	<0.001
TG (mmol/L)	0.5 (0.1)	0.6 (0.3)	0.8 (0.3)	1.0 (0.4)	<0.001
HDL (mmol/L)	1.4 (0.3)	1.3 (0.3)	1.2 (0.3)	1.0 (0.3)	<0.001
Glucose (mmol/L)	4.3 (0.3)	4.6 (0.5)	4.8 (0.5)	4.9 (0.4)	<0.001
SBP (mmHg)	97.3 (5.4)	99.0 (6.8)	101.0 (8.5)	104.6 (8.6)	<0.001
**Overweight status (%)**					<0.001
Overweight	3.9	7.9	9.8	27.9	
Obese	0.2	0.3	3.3	17.4	
**Fat %**	22.7 (5.5	23.6 (6.0	24.2 (6.2	28.8 (6.2	<0.001
**BMI (kg/m** ^**2**^ **)**	15.1 (1.1)	15.4 (1.3)	15.8 (1.6)	17.2 (2.3)	<0.001

WHtR = Waist to height ratio, HDL = High-density lipoprotein cholesterol, TG = triglycerides, SBP = Systolic blood pressure, PEP = pre-ejection period, HR = Heart rate, RSA = Respiratory Sinus Arrhythmia, CAB = Cardio Autonomic Balance.

### Associations between ANS and continuous metabolic components

After adjustments for confounders (model 2) increased HR was significantly associated with increasing WHtR*100 (β = 0.226;95%CI:0.069to0.382), SBP (β = 1.68;1.30to2.06) as well as with the sum score (β = 0.34;0.19to0.48; Table 2). RSA was negatively associated with WHtR*100 (β = -0.036;-0.058to-0.013), SBP (β = -0.11;-0.17to-0.06) and the sum score (β = -0.030;-0.051to-0.010). PEP was negatively associated with SBP (β = -0.60;-1.00to-0.21). CAB was negatively associated with WHtR (β = -0.113;-0.209to-0.018) and SBP (β = -0.54;-0.77to-0.31) and CoAR only with the sum score (β = -0.11;-0.21to-0.005). The explained variance of the total models was generally low; however, the highest was for SBP (10%), followed by WHtR (∼9%) and with significant contributions of child’s sex, age, height (only SBP) and ethnicity.

**Table 2:** Associations between autonomic nervous system activity and continuous metabolic components at age 5–6 years.

	WHtR x 100		SBP (mmHg)		HDL (mmol/L)		TG (ln) (mmol/L)		Glucose (mmol/L)		Adverse metabolic profile ^†^	
	ß	*R* ^*2*^	ß	*R* ^*2*^	ß	*R* ^*2*^	ß	*R* ^*2*^	ß	*R* ^*2*^	ß	*R* ^*2*^
**HR (per 10 beats/min)**												
Model 1	0.226**	0.04	1.68***	0.12	0.002	0.02	0.005	0.03	0.023	0.03	0.342***	0.02
Model 2	0.158**	0.09	1.60***	0.13	0.002	0.02	0.006	0.04	0.021	0.06	0.313***	0.03
**RSA (per 10 ms)**												
Model 1	-0.036**	0.04	-0.11***	0.08	-0.001	0.02	-0.001	0.03	-0.003	0.03	-0.030**	0.01
Model 2	-0.031**	0.09	-0.10***	0.10	-0.001	0.02	-0.001	0.04	-0.002	0.06	-0.027**	0.03
**PEP per 10 ms)**												
Model 1	0.071	0.03	-0.60**	0.08	-0.006	0.02	0.001	0.03	0.008	0.03	-0.026	0.01
Model 2	0.018	0.09	-0.42*	0.09	-0.006	0.02	0.001	0.03	0.005	0.04	-0.016	0.02
**CAB (per 1 U)**												
Model 1	-0.113*	0.04	-0.54***	0.09	-0.004	0.02	-0.001	0.03	-0.006	0.03	-0.102	0.01
Model 2	-0.100*	0.09	-0.43***	0.10	-0.004	0.02	-0.002	0.03	-0.005	0.06	-0.084	0.03
**CoAR per 1 U)**												
Model 1	-0.111*	0.04	-0.12	0.09	-0.000	0.02	-0.004	0.03	-0.013	0.03	-0.101*	0.01
Model 2	-0.100	0.09	-0.16	0.10	-0.000	0.02	-0.005	0.03	-0.013	0.03	-0.111*	0.03

Note. The residuals from all models were normally distributed and variances were homogeneous. The R^2^ represents the unadjusted explained variance.

Model 1: adjusted for age, sex and time of the day of measurement (when SBP or total metabolic profile were the outcome, additional adjustment for child’s height)

Model 2: Model 1 + education level, ethnicity, sports participation, screen time, hours of sleep and anxiety.

WHtR = Waist to height ratio, HDL = High density lipoprotein cholesterol, TG = triglycerides, SBP = Systolic blood pressure, PEP = pre-ejection period, HR = Heart rate, RSA = Respiratory Sinus Arrhythmia, CAB = Cardio-autonomic balance, CoAR = cardiac autonomic regulation.

^†^ Defined as sum of z-scores of WHtR, SBP, TG, -1xHDL, Glucose

* p<0.05;

** p<0.01;

*** p<0.001.

### Associations between ANS and dichotomous metabolic components (as risk factors)

**Fig 2:**
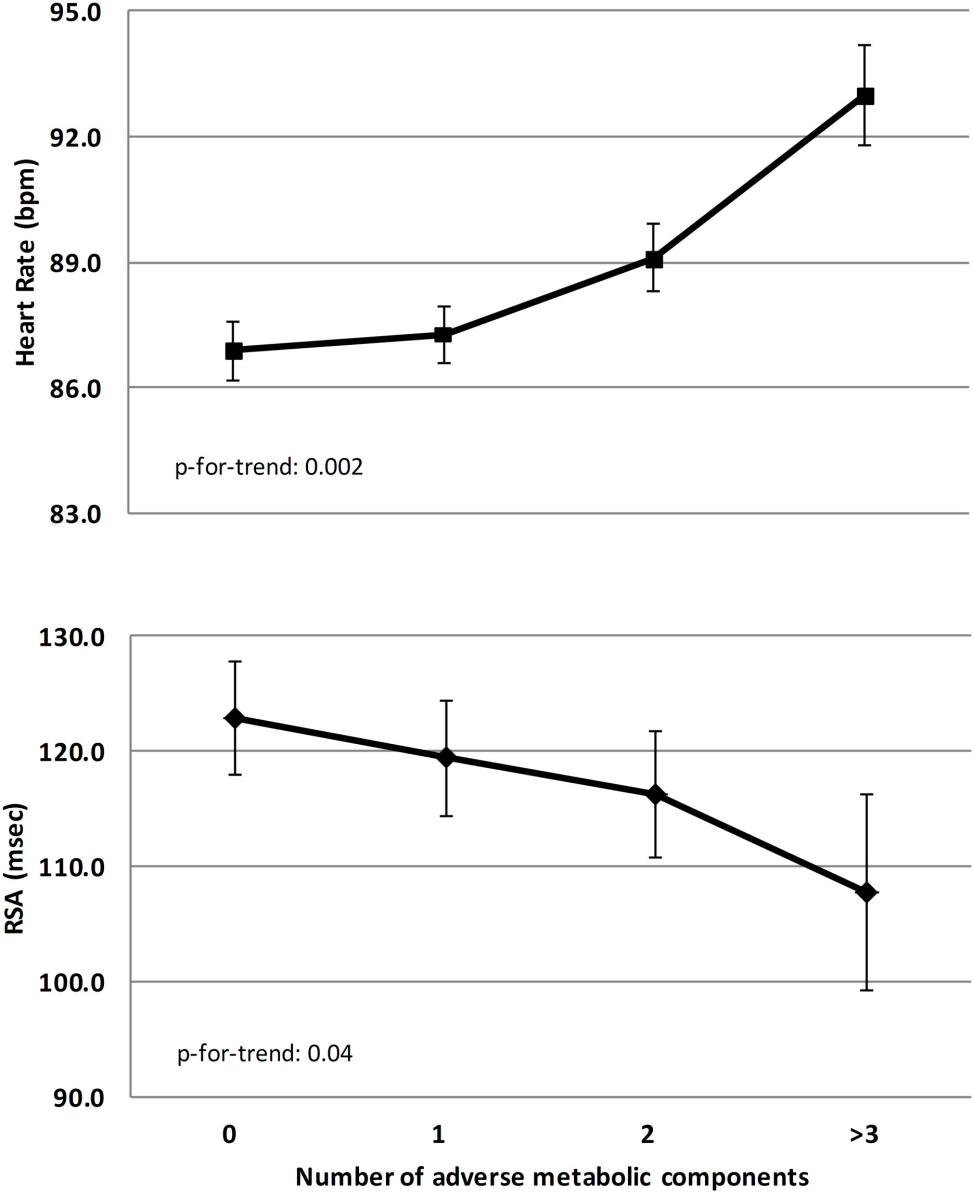
Mean HR and RSA as a function of the number of adverse metabolic components (high waist-height ratio, high glucose, high triglycerides, prehypertension and low HDL). Results are adjusted for age, sex, time of the day of measurement, education level, ethnicity, sports participation, screen time, hours of sleep and anxiety.

When risk factors were examined (Table 3), after adjustments for confounders (model 2) increased HR (per 10 beats/min) was associated with an increased risk of a high WHtR (OR = 1.37;95%CI:1.08to1.72), pre-hypertension (OR = 1.80;1.48–2.18). Increased RSA (per 10 msec) was associated with a lower risk for high WHtR (OR = 0.95;0.91–0.99) and pre-hypertension (OR = 0.97;0.94to1.00). Increased CoAR was only associated with decreased risk for high WHtR (OR = 0.78;0.65to0.93). The association of HR and RSA with the number of adverse metabolic components is presented in Fig 2; this shows that increasing HR and decreasing RSA were associated with an increased number of adverse metabolic components. This resulted in an increased risk for adverse clustering of metabolic components (≥ 3 out of 5 present); however, this was only significant for increased HR (per 10 beats/min: OR = 1.52; 1.17–1.98; Table 3). CAB and PEP were not associated with an increased risk for adverse clustering of metabolic profile or its individual components.

**Table 3:** Associations between autonomic nervous system activity and risk for adverse metabolic components at age 5–6 years.

	High WHtR		Pre-hypertension		Low HDL		High TG		High Glucose		Adverse metabolic profile ^†^	
	OR	*95%CI*	OR	*95%CI*	OR	*95%CI*	OR	*95%CI*	OR	*95%CI*	OR	*95%CI*
**HR (per 10 beats/min)**												
Model 1	1.38	1.10;1.72**	1.82	1.51;2.20***	0.90	0.79;1.03	1.10	0.97;1.25	1.02	0.91;1.16	1.52	1.20;1.93***
Model 2	1.37	1.08;1.72**	1.80	1.48;2.18***	0.91	0.79;1.05	1.11	0.97;1.26	1.02	0.90;1.15	1.56	1.21;2.00***
**RSA (per 10 ms)**												
Model 1	0.95	0.92;0.99*	0.97	0.94;1.00*	0.99	0.97;1.01	0.99	0.97;1.01	1.00	0.98;1.02	0.98	0.94;1.01
Model 2	0.95	0.91;0.99*	0.97	0.94;1.00*	0.99	0.97;1.01	0.99	0.97;1.01	1.00	0.98;1.02	0.97	0.93;1.01
**PEP per 10 ms)**												
Model 1	1.14	0.90;1.49	0.96	0.79;1.17	0.99	0.86;1.15	0.98	0.86;1.12	1.06	0.92;1.20	1.05	0.81;1.36
Model 2	1.16	0.91;1.49	0.99	0.81;1.22	0.98	0.85;1.14	0.98	0.86;1.12	1.06	0.93;1.21	1.05	0.80;1.36
**CAB (per 1 U)**												
Model 1	0.94	0.81;1.09	0.91	0.81;1.03	0.97	0.89;1.06	0.97	0.90;1.05	1.03	0.95;1.11	0.97	0.83;1.13
Model 2	0.94	0.80;1.09	0.93	0.82;1.05	0.97	0.89;1.05	0.97	0.90;1.05	1.03	0.95;1.11	0.96	0.81;1.12
**CoAR per 1 U)**												
Model 1	0.79	0.67;0.94**	0.92	0.81;1.06	0.97	0.88;1.07	0.98	0.89;1.07	0.98	0.90;1.07	0.91	0.76;1.09
Model 2	0.78	0.65;0.93**	0.91	0.79;1.05	0.97	0.88;1.07	0.98	0.89;1.08	0.98	0.90;1.07	0.90	0.75;1.08

Note. Goodness-of-fit tests showed no evidence of lack of fit (P>0.23). WHtR = Waist to height ratio, HDL = High density lipoprotein cholesterol, TG = triglycerides, SBP = Systolic blood pressure, PEP = pre-ejection period, HR = Heart rate, RSA = Respiratory Sinus Arrhythmia, CAB = Cardiac autonomic balance, CoAR = cardiac autonomic regulation.

Model 1: adjusted for age, sex and time of the day of measurement (when SBP or total metabolic profile were the outcome, additional adjustment for child’s height)

Model 2: Model 1 + education level, ethnicity, sports participation, screen time, hours of sleep and anxiety

^†^ Defined as 3 or more out of 5 adverse components

* p<0.05;

** p<0.01;

*** p<0.001.

## Discussion

In this large community-based and overall healthy cohort of children aged 5–6 years, we found that decreased parasympathetic activity was associated with a less favorable metabolic profile, mainly driven by adverse associations with higher WHtR and SBP. Our observations are in line with comparable observations made in adults[8,9]. Therefore, our findings suggest that a decreased parasympathetic activity in association with a more pronounced metabolic profile is already determined from early childhood on.

An increase in HR and a decrease in RSA were associated with increased WHtR, SBP, and the sum of the separate metabolic components. These results are comparable to those of Zhou et al. who measured components of the metabolic syndrome in a selected group of 180 Chinese children[17]; they found a decrease in several measures of HRV with an increasing number of metabolic risk factors. In accordance with our study, HRV measures that reflected parasympathetic activity were significantly associated with the amount of fat (such as waist circumference and BMI) and SBP[17]. Others who compared obese vs. non-obese children also found decreased parasympathetic activity and increased HR in obese children[18,19,21,25].

So far most studies in children used the ratio between low frequency domain (LF) and high frequency domain (HF) indices of HRV as a reflection of ANS (or sympathicovagal) balance. Most studies found an increased LF/HF ratio in obese children[19,21,25] or a positive association with BMI[20] or fat percentage[22], whereas some did not confirm this finding[17]. Our observations (including measurement of HR, PEP CAB and CoAR) suggest that this shift in ANS balance at a young age is more a result of decreased parasympathetic activity rather than of increased sympathetic activity.

It is established that increased sympathetic activity is closely associated with SBP in both childhood[17] and adulthood [8,14] as also shown by the positive association between PEP and SBP in our study. We found no association between PEP and any of the other metabolic components, which is in line with a previous observation by Yakinci et al. who found no difference in sympathetic activity (with use of orthostatic testing) and observed reduced parasympathetic activity in a clinical study comparing 33 obese and 30 non-obese children.[23]. In adults, most studies have also reported significant associations between increased sympathetic activity and metabolic aberrations[7,8,10], This may suggest a shift towards increased sympathetic dominance with advancing age. The mechanisms linking metabolic function with ANS activation are complex and the cause-effect relationships are still not completely understood. However, it is suggested that reduced parasympathetic activity and increased sympathetic activity predict weight gain and obesity. Graziano et al. followed 268 children from age 5.5. years to 10.5 years and found increased weight gain and increased risk of overweight at age 10.5 years in children with lower RSA at age 5.5 years[25]. Reduced vagal tone is associated with more increase in visceral fat and increased inflammatory response[11]. At adult age, decreased parasympathetic activity and increased sympathetic activity are strong predictors for type II diabetes[7,15,16] and hypertension[10,14]; in addition, various studies in animals and human adults showed that vagal stimulation had a positive effect on satiety and food intake[10,41]. These results argue for a role of disturbed ANS in the etiology of obesity, metabolic disturbances and hypertension. Additionally, visceral fat secretes hormone like components, such as free fatty acids and adipokines, like leptin, causing insulin resistance, and all are able to increase sympathetic activity[7,10,12]. Against this background, we hypothesize that parasympathetic activity is more prominent as a metabolic actor in metabolism at a healthy young age and that, along with ageing, visceral fat accumulates and hepatic insulin resistance increases with a subsequent change towards a more sympathetic dominance. Hence, a vicious circle is established and our results suggest that this might start early in life.

### Strengths and limitations

It should be noted that our included children were relatively healthy, as only 10% of our young population fits into the definition of childhood overweight (including obesity) and only 5.6% had an adverse clustering of the metabolic profile. Moreover, we measured our children during resting conditions and several studies indicate that ANS dysregulation becomes more apparent specifically during stress or a metabolic challenge[1,13,25]. Therefore, it would be valuable to investigate different patterns of ANS reactivity in relation to the child’s metabolic profile to gain more knowledge about early aberrations. Due to the cross-sectional design of the present study no causality can be shown and the results should be interpreted with some caution. A major strength of our study is the large sample size, a community-based sample and the combination of multiple ANS measures. Because our ANS measurements were taken outside the laboratory environment, we were able to include a large number of subjects. RSA is considered a reliable estimate for indicating parasympathetic activity as it rules out variability in HR due to respiratory influences[42] which are not induced by vagal activity. Especially in children, other time domain measures like RMSSD or SDNN or frequency domain measures, like LF and HF, could be biased by variations in respirations which are difficult to standardise. PEP is a valid marker of sympathetic activity, often used in adult populations but also in pediatric populations[42,43]. It is known that stability in PEP, HR and RSA is moderate to high, implying that it can be used to detect real differences in children[44]. Measurements in our children during both lying and sitting positions showed high correlations, indicating a high level of short-term stability.

Unfortunately, as in most cohort studies, selective loss to follow-up is present. The current subgroup was slightly healthier than the non-response group and, therefore, the associations could be an underestimation of the effects found in the general population. Another strength of our study is that we used fasting blood samples to avoid differences in glucose, HDL-cholesterol and TG levels due to recent food intake. We adjusted for ethnicity, sleep duration, general anxiety, amount of screen time and sport participation, all of which are related to ANS activity and metabolic function; this was also clearly seen in our study (Table 1). One might also argue that this is overcorrection as these factors could (in part) be related to adverse metabolic function via altered ANS activity. Moreover, the explained variance of the full models was at the most 13% (SBP) and, in general, child’s sex, age and ethnicity contributed the most. This means that variations in the metabolic outcome measures were largely unexplained at this young age. More research on other explanatory factors is needed, as well as on the development of these metabolic factors as children become older.

## Conclusions

This study confirms an association between the ANS balance and metabolic profile, in line with earlier results in adult populations. Indeed, a decreased parasympathetic activity is associated with central adiposity and higher SBP, indicative of increased metabolic risk, already at age 5–6 years. Against the background of our results, we hypothesize that adapting the ANS balance by specific interventions, e.g. physical activity programs or stress-reducing programs, already during early childhood could prevent obesity and its associated metabolic profile later in life. Our results needs to be confirmed by additional studies in child populations along the trajectory of the children’s neurodevelopment.

## Supporting Information

S1 Table Non-response analysis (n = 6119). The non-response group consisted of children who were eligible for the study (approached for the 5-year measurement round without congenital conditions affecting the cardiovascular system or the autonomic nervous system or using medication influencing the autonomic nervous system), but were not included.
(DOC)
