# Supplementary material for: Cardiac Autonomic Nervous System Activation and Metabolic Profile in Young Children: The ABCD Study
Source: PLoS One. 2015 Sep 22;10(9):e0138302. doi: 10.1371/journal.pone.0138302 (PMC4579089; doi:10.1371/journal.pone.0138302)
Supplement: S1 Table — The non-response group consisted of children who were eligible for the study (approached for the 5-year measurement round without congenital conditions affecting the cardiovascular system or the autonomic nervous system or using medication influencing the autonomic nervous system), but were not included. (DOC) [file pone.0138302.s001.doc]

Table S1: Non-response analysis (n=6119)^*^

|  |  |  |  |  |  |
| --- | --- | --- | --- | --- | --- |
|  |  | **Response (n=1540)** | **Non response (n=4579)** |  |  |
|  | **Characteristics** | **Mean/% (SD)** | **Mean/% (SD)** | ***p-value*** |  |
|  | Girls (%) | 49.4 | 50.4 | 0.521 |  |
|  | Gestational age (weeks) | 39.9 (1.6) | 39.8 (1.8) | 0.117 |  |
|  | Birth weight (grams) | 3511 (537) | 3434 (552) | <0.001 |  |
|  | Years after primary school mother | 9.8 (3.7 | 8.8 (4.0) | <0.001 |  |
|  | Age mother (at first prenatal screening) | 32.3 ( 4.6 | 30.6 (5.2 | <0.001 |  |
|  | Smoking during pregnancy (% yes) | 9.0 | 10.0 | 0.247 |  |
|  | Ethnicity mother (%) |  |  | <0.001 |  |
|  | Dutch | 75.8 | 66.9 |  |  |
|  | Turkish | 2.3 | 3.7 |  |  |
|  | Moroccan | 4.2 | 6.7 |  |  |
|  | Surinamese | 3.5 | 5.5 |  |  |
|  | Other non-Western | 8.1 | 9.9 |  |  |
|  | Other Western | 6.1 | 7.2 |  |  |

^*^ The non-response group consisted of children who were eligible for the study (approached for the 5-year measurement round without congenital conditions affecting the cardiovascular system or the autonomic nervous system or using medication influencing the autonomic nervous system), but were not included.
